# CircMMP11 regulates proliferation, migration, invasion, and apoptosis of breast cancer cells through miR-625-5p/ZEB2 axis

**DOI:** 10.1186/s12935-021-01816-z

**Published:** 2021-02-25

**Authors:** Liqiang Qi, Bo Sun, Beibei Yang, Su Lu

**Affiliations:** 1 Department of Breast Surgical Oncology, Cancer Institute and Cancer Hospital, Chinese Academy of Medical Sciences and Peking Union Medical College, No. 17 Panjiayuan Nanli, Chaoyang District, Beijing, 100021 China; 2 The 2nd Department of Breast Cancer, Tianjin Medical University Cancer Institute and Hospital, Tianjin, China

**Keywords:** circRNA, circMMP11, miR-625-5p, ZEB2, BC

## Abstract

**Background:**

Circular RNAs (circRNAs) have been demonstrated to play significant roles in regulating gene expression in tumorigenesis, including breast cancer (BC). This study was designed to explore the role and underlying molecular mechanisms of circMMP11 in BC.

**Methods:**

The real-time quantitative polymerase chain reaction (RT-qPCR) assay was used for examining expression of circMMP11, microRNA-625-5p (miR-625-5p), and Zinc finger E-box binding homeobox-2 (ZEB2). The protein expression of ZEB2, Vimentin, and E-cadherin was assessed by western blot assay. The proliferation ability of BC cells was determined by 3-(4,5-dimethylthiazol-2-yl)-2,5-diphenyl-2*H*-tetrazol-3-ium bromide (MTT) and colony-forming assays. The transwell assay was used to measure migration and invasion of BC cells. The apoptotic cells were examined by flow cytometry assay. The interaction association among circMMP11, miR-625-5p, and ZEB2 was confirmed by RNA pull-down and dual-luciferase report assays. A xenograft experiment was established to clarify the role of circMMP11 silencing in vivo.

**Results:**

We found that circMMP11 and ZEB2 were overexpressed in BC tissues and cells compared with controls. The suppression of circMMP11 or ZEB2 repressed proliferation, migration, and invasion while induced apoptosis of BC cells. Additionally, miR-625-5p, interacted with ZEB2, was a target of circMMP11 in BC cells. CircMMP11 regulated the expression of ZEB2 by targeting miR-625-5p. Knockdown of circMMP11-mediated effects on BC cells could be abolished by overexpression of ZEB2. Consistently, silencing of circMMP11 impeded the tumor growth in vivo.

**Conclusions:**

CircMMP11/miR-625-5p/ZEB2 axis affected proliferation, migration, invasion, and apoptosis of BC cells through the mechanism of competing endogenous RNAs (ceRNA), indicating that circMMP11 was an oncogenic circRNA in BC.

## Introduction

Breast cancer (BC) is a common female cancer all over the world [1]. The morbidity of BC has been rising, over 2 million females were diagnosed with BC according to the statistic [2]. Although improved prognosis of patients with BC had achieved, BC remains leading reason of cancer mortality among females [3]. Therefore, it is meaningful to look into pathogenesis and find diagnosis biomarkers of BC.

Circular RNAs (circRNAs), a group of RNA molecules with closed covalent loops, play critical roles in initiation and development of the multiple malignancies [4]. Accumulating evidence has revealed multiple characterization of circRNAs, including microRNA sponges [5], CircMMP11 (hsa_circ_0062558) is derived from the Matrix Metalloprotease 11 (MMP11) gene and located on chr22 (24,125,597–24,126,503). A previous research reported that a series of differentially expressed circRNAs could function as novel biomarkers for BC, including circMMP11 [6]. Interestingly, circMMP11 could function as a competitive endogenous RNA (ceRNA) for miR-1204 to participate in the development of BC, indicating that circMMP11 was a potential therapeutic target for BC [7].

MicroRNAs (miRNAs) could function as an important class of posttranscriptional regulators through base complementation with the 3′untranslated region (3′UTR) of mRNAs, thereby altering the gene expression of carcinogenic or tumor-inhibitory genes [8]. For example, miR-625-5p was reported to play significance roles in malignant tumors. The upregulation of miR-625-5p restricted cell growth of gastric cancer cells [9]. Additionally, the tumor-suppressive function also was reported in BC; downregulation of miR-625 was found in BC tissues and cells, which was closely related to poor clinical outcomes of BC patients [10]. Insufficiently, the functions of miR-625-5p are not deeply investigated in BC.

The transcriptional factor Zinc finger E-box binding homeobox-2 (ZEB2), belonging to ZEB family, is implicated in some signaling pathways involved in epithelial–mesenchymal transition (EMT) process [11]. Not surprisingly, ZEB2 was reported to be essentially involved in EMT process of BC cells [12]. As previous report, the metastasis-associated function of ZEB2 has been demonstrated in multiple tumors, including BC [13]. Analogously, Chen et al. [14] also found that miR-30a inhibited migration and invasion of nasopharyngeal carcinoma cells by modulating ZEB2.

Therefore, this research was designed to inspect the expression and regulatory mechanisms of circMMP11 in BC. Based on above researches, we hypothesized that circMMP11 was involved in BC process via regulatory networks of ceRNA.

## Materials and methods

### Patient specimens

We collected 34 paired BC specimens and the peracancer tissues from BC patients with surgical tumor resections at Cancer Institute and Cancer Hospital, Chinese Academy of Medical Sciences and Peking Union Medical College. All recruited BC patients completed the written informed consent. The removed tissues were stored at − 80 °C after freeze in liquid nitrogen. The entire investigation was authorized by the Ethics Committee of Cancer Institute and Cancer Hospital, Chinese Academy of Medical Sciences and Peking Union Medical College. The clinicopathologic features of BC patients were presented in Table 1. BC patients were selected for further investigation based on two strict criteria: (1) patients were first diagnosed with BC; (2) patients provided the written informed consent. Exclusion criteria: (1) patients received neoadjuvant therapy; (2) patients with other complicated diseases.

**Table 1:** Correlation of clinicopathological features of BC patients with circMMP11expression levels

Characteristics	All cases	circMMP11 expression		*p* value
		High	Low	
Age				0.7283
≥ 45	20	9	11	
< 45	14	8	6	
Tumor				0.7319
Left	18	10	8	
Right	16	7	9	
Tumor size (cm)				0.1571
≥ 2	13	9	4	
< 2	20	8	12	
Molecular subtype				0.2962
Luminal A	12	7	5	
Luminal B	13	6	7	
HER2E	2	2	0	
TNBC	7	2	5	
Family history of breast cancer				0.4905
Yes	15	9	6	
None	19	8	11	
Lymph node metestasis				0.0039**
Negative	21	6	15	
Positive	13	11	2	
TNM stage				0.0366*
I–II	15	4	11	
III–IV	19	13	6	

*HER2E* Her2-enriched, *TNBC* Triple Negative Breast Cancer

*P < 0.05, **P < 0.01, statistically significant

### Cell lines and cell culture

The human normal breast epithelial cells (MCF-10A; basal subtype) and BC cell lines (MCF-7; Luminal A subtype and MDA-MB-231; basal subtype) were purchased from the American Type Culture Collection (Rockville, MD, USA). Dulbecco’s modified Eagle medium (Biochrom KG, Berlin, Germany) contained with 10% (v/v) fetal bovine serum (FBS; Sigma, San Francisco, CA, USA) and 1% penicillin/streptomycin (Sigma) was used to incubate these cells in under standard culture conditions (5% CO_2_, 37 °C). In addition, MCF-7 cells were cultured according to ATCC recommendation that 0.01 mg/mL human recombinant insulin was used to make the complete growth medium.

### Real-time quantitative polymerase chain reaction (RT-qPCR) assay

Total RNA was extracted by RNA extraction kit (Thermo Fisher Scientific, Waltham, MA, USA) in the light of the user’s guideline. Following, total RNA was converted into complementary DNA (cDNA) by cDNA synthesis kit II (Exiqon, Woburn, MA, USA) or TransScript miRNA First-Strand cDNA Synthesis SuperMix (Transgen biotech, Beijing, China). The RT-qPCR assay was conducted under A Roche Light-Cycler (Roche, Basel, Switzerland) with QuantiTect SYBR Green RT-PCR Kit (Qiagen, Dusseldorf, NRW, Germany). In addition, endogenous small nuclear RNA U6 was used as the internal reference for miR-625-5p, while glyceraldehyde phosphate dehydrogenase (GAPDH) was used as the internal control for circMMP11 and ZEB2 based on the 2^−ΔΔCt^ method. The primers were shown as follows:

- circMMP11 (up 5′-CTTTTCGCAGCACTGCTATCC-3′; down 5′-CCTTCCAGAGCCTTCACCTT-3′);
- miR-625-5p (up 5′-GCCGAGAGGGGGAAAGTTCTA-3′; down, 5′-CAGTGCAGGGTCCGAGGTAT-3′);
- ZEB2 (up 5′-CAAGAGGCGCAAACAAGCC-3′; down 5′-GGTTGGCAATACCGTCATCC-3′);
- GAPDH (up 5′-GTGTTCCTACCCCCAATGTG-3′; down 5′-CATCGAAGGTGGAAGAGTGG-3′);
- U6 (up 5′-ATCCTTACGCACCCAGTCCA-3′; down 5′-GAACGCTTCACGAATTTGC-3′).

### Western blot assay

Briefly, lysis buffer (Cell Signaling Technology, Danvers, MA, USA) was rapidly added to cells or tissues after washing by pre-cooled phosphate buffer saline. After quantifying and boiling, 40 µg of total protein was subject to 10% sodium dodecyl sulfate polyacrylamide gel electrophoresis and then transferred nitrocellulose membranes (GE Healthcare, Piscataway, NJ, USA). After blocking by 5% non-fat milk for 2 h, membranes were interacted with primary anti-bodies overnight at 4 °C, including anti-E-cadherin (#3195S; 1:1500 dilution), anti-Vimentin (#5741S; 1:1500 dilution), anti-ZEB2 (#3396S; 1:1500 dilution), and anti-β-actin (#4970S; 1:1500 dilution; all purchased from Cell Signaling Technology). Following, membranes were reacted to HRP-conjugated secondary antibody (#7074S; 1:200 dilution; Cell Signaling Technology). The relative expression of proteins was detected under ChemiDoc MP imaging system (Bio-Rad, Hercules, CA, USA) by Clarity™ Western ECL Substrate Kit (Bio-Rad).

### Transfection assay

Small interfering RNA (siRNA) targeting circMMP11 was amplified and synthesized by GenePharma (Shanghai, China). The sequences of si-circMMP11#1 were: 5′-AAGAAGAAGGUUUAUACACAC-3′; si-circMMP11#2, 5′-AGAAGAAGAAGGUUUAUACAC-3′; si-circMMP11#3; 5′-AAAACAACUGUGUUUAAUGAC-3′; Negative siRNA: 5′-GGCCUAAAGUAGUAGCUAUTT-3′; ZEB2-overexpression vector (ZEB2) was accomplished by amplifying cDNA of ZEB2 and subcloning into the pcDNA vector (GenePharma). MiR-625-5p mimic (miR-625-5p), miR-NC, miR-625-5p inhibitor (in-miR-625-5p), in-miR-con were purchased from Sangon (Shanghai, China). The oligonucleotides and vectors were transferred into BC cells by Lipofectamine 2000 (Thermo Fisher Scientific).

### 3-(4,5-Dimethylthiazol-2-yl)-2,5-diphenyl-2*H*-tetrazol-3-ium bromide (MTT) assay

The cell viability of BC cells was determined by MTT assay. Briefly, the transfected BC cells were tiled to a 96-well plate (3000 cells/well). Following, 20 µL of MTT solution (Sigma) was added into cells and allowed to incubate for indicate times. After 4 h, the dimethyl sulfoxide (Sigma) was used to dissolve formazan crystals. The absorbance was measured under the multi-well scanning spectrophotometer (Bio-Rad; wavelength: 450 nm). Furthermore, the colony-forming assay was performed as previous description [15].

### Transwell assay

Approximately 5 × 10^4^ MCF-7 or MDA-MB-231 cells in serum-free medium were added into upper chamber of 24-well transwell chamber (8-µm pore size; BD Biosciences, San Jose, CA, USA), and the lower chamber was filled with complete medium containing 10% FBS. After 24 h, cells on the basal side of the membrane were stained by 0.1% crystal violet (Sigma) and photographed under the microscope (Leica, Wetzlar, Germany; 100× magnification). For invasion assay, upper chamber was additionally covered with matrigel (BD Biosciences).

### Cell apoptosis assay

Transfected BC cells were collected by trypsin, washed with pre-cooled phosphate buffer saline, and then re-suspended in Binding Buffer containing Annexin V labeled with fluorescein isothiocyanate (FITC) and propidium iodide (PI) (BestBio, Shanghai, China). Followed by incubated at 4 °C for 30 min, the apoptotic cells were determined on flow cytometric (Attune, Life Technologies, Darmstadt, Germany).

### RNA pull‐down assay

The biotin-labeled RNA probe (Bio-circMMP11 probe) was synthesized by GenePharma. MCF-7 or MDA-MB-231 cells were lysed by lysis buffer (Cell Signaling Technology). Subsequently, 100 µL of cell lysates was incubated with Bio-circMMP11 probe at 4 °C for 4 h, with Bio-con as control, and the treated with 50 µL of streptavidin beads (Cell Signaling Technology) at 4 °C overnight to generate probe-bound dynabeads. The RNA complexes were extracted and measured by RT-qPCR assay. Similarly, the 3′end biotinylated miR-625-5p (Riboio, Bio-miR-625-5p probe) was used to pull-down circMMP11 and ZEB2 in cell lysates.

### Dual‐luciferase report assay

Based on bioinformatics prediction starbase (http://starbase.sysu.edu.cn/), Circbank (http://www.circbank.cn/), and Circular RNA interactome (https://circinteractome.irp.nia.nih.gov/), miR-625-5p was selected as a candidate target of circMMP11. The complementary sequences between miR-625-5p and ZEB2 were presented by Targetscan (http://www.targetscan.org/vert_72/). Partial sequences of circMMP11 and 3′UTR of ZEB2 that contain the putative binding sites for miR-625-5p were synthesized and cloned into luciferase report vectors (Ambion, Foster City, CA, USA). Subsequently, the BC cells were co-transfected with miR-625-5p mimic or miR-NC and the wild or mutant type reports. The relative firefly luciferase activity was determined under the VICTOR2 fluorometry (PerkinElmer, Waltham, MA, USA) and normalized to Renilla luciferase activity.

### Animal experiment

Six-week-old female BALB/c nude mice (Vital River Laboratory, Beijing, China) were housed under specific pathogen-free conditions. MCF-7 stably transfected with sh-circMMP11 (GeneCopoeia, Rockville, MD, USA) were inoculated into right back of nude mice (5 × 10^7^ cells/mouse in 200 µL of complete growth medium; N = 6), with sh-NC as control. The xenograft volume was examined with digital calipers according to the volume = 1/2 (length × width^2^). All of our animal experimental protocols had been approved by the Institutional Animal Care and Use Committee of Cancer Institute and Cancer Hospital, Chinese Academy of Medical Sciences and Peking Union Medical College. The sh-circMMP11 sequence were amplified and then cloned into pLKO.1-Puro vector (GeneCopoeia) between AgeI and EcoRI sites. Specific short hairpin RNA against circMMP11 (sh-circMMP11; sense strand 5′-ccggAGAAGAAGAAGGTTTATACACttcaagagaGTGTATAAACCTTCTTCTTCTTTTTTTGGTACC-3′. antisense strand 5′-aattGGTACCAAAAAA AGAAGAAGAAGGUUUAUACACTCTCTTGAAGTGTATAAACCTTCTTCTTCT-3′). In addition, ccgg and ttaa was used to generate sticky-end by AgeI and EcoRI, and ttcaagaga was used as stem-loop sequence.

### Statistical analysis

All data were shown as mean ± standard deviation and performed in triplicate. Comparison of two treatment groups was conducted by Student’s *t*-test, while significant difference of multiple groups was assessed by one-way analysis of variance. Statistical analysis was conducted by GraphPad Prism 7 (GraphPad, La Jolla, CA, USA), and significant difference was considered if *P*-value less than 0.05. Pearson’s correlation analysis was used to reveal correlation relationship among circMMP11, miR-625-5p, and ZEB2 in BC tissues.

## Results

### CircMMP11 and ZEB2 were overexpressed in BC tissues and cells

**Fig. 1:**
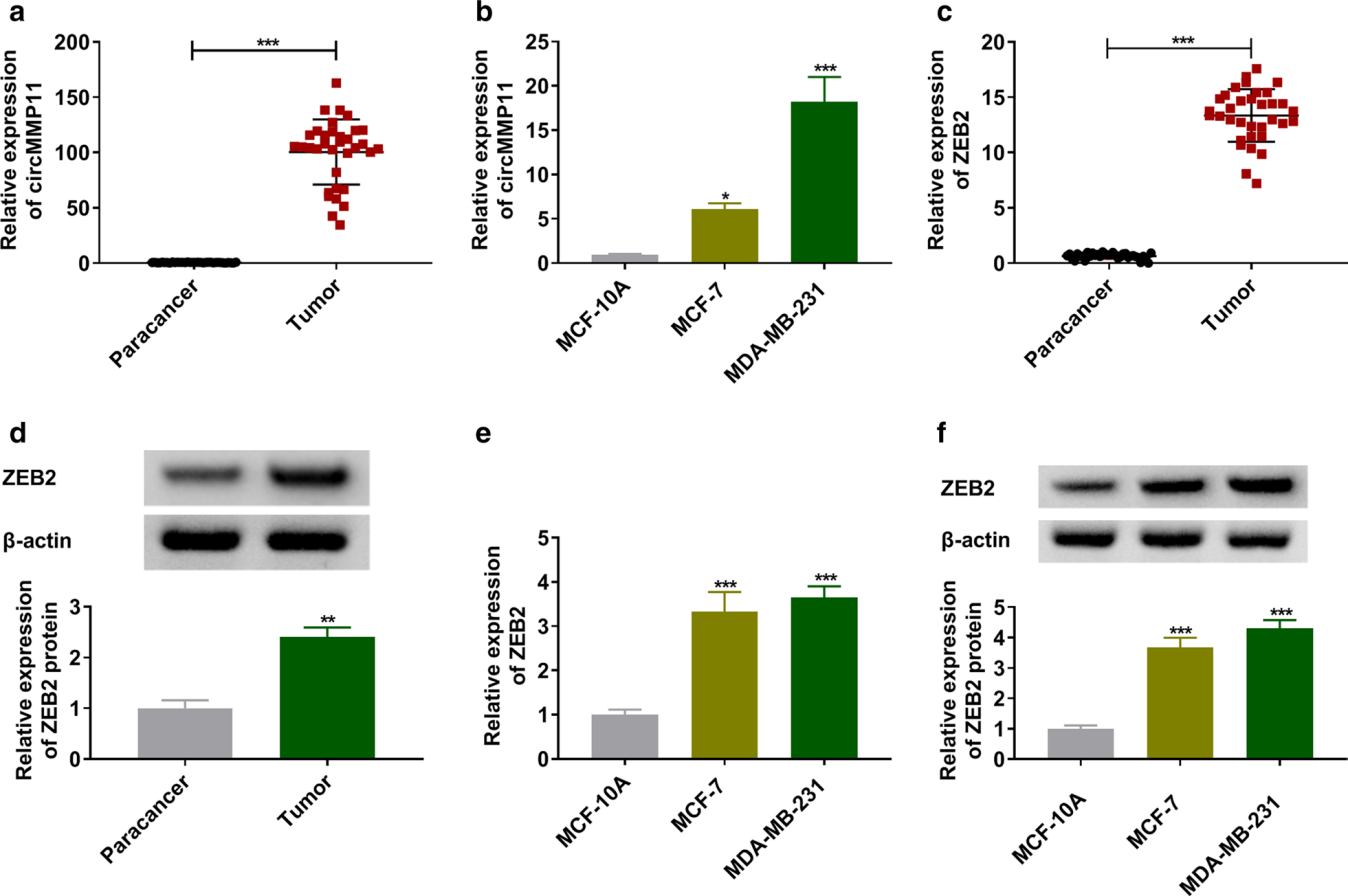
The expression levels of circMMP11 and ZEB2 in breast cancer tissues and cells. **a**, **b** The relative expression level of circMMP11 was determined by RT-qPCR assay in breast cancer tissues and cells, along with in matched controls. **c**–**f** The RT-qPCR and western blot assays were performed to assess the expression of ZEB2 in breast cancer tissues and cells. *P < 0.05, **P < 0.01, ***P < 0.001

To explore the role of circMMP11 in BC, the expression level of circMMP11 was quantified. The results of RT-qPCR data suggested that circMMP11 were overexpressed in BC tissues and cells when compared with paracancer tissues and MCF-10A cells (Fig. 1a, b). Furthermore, ZEB2 also was significantly upregulated in BC tissues compared with adjacent normal tissues (Fig. 1c, d). The analogous results were confirmed in BC cells that MCF-7 and MDA-MB-231 cells showed the relative higher expression of ZEB2 than MCF-10A cells (Fig. 1e, f). We hypothesized that circMMP11 and ZEB2 played key roles in BC.

### Knockdown of circMMP11 constrained proliferation, migration, and invasion while induced apoptosis of BC cells

**Fig. 2:**
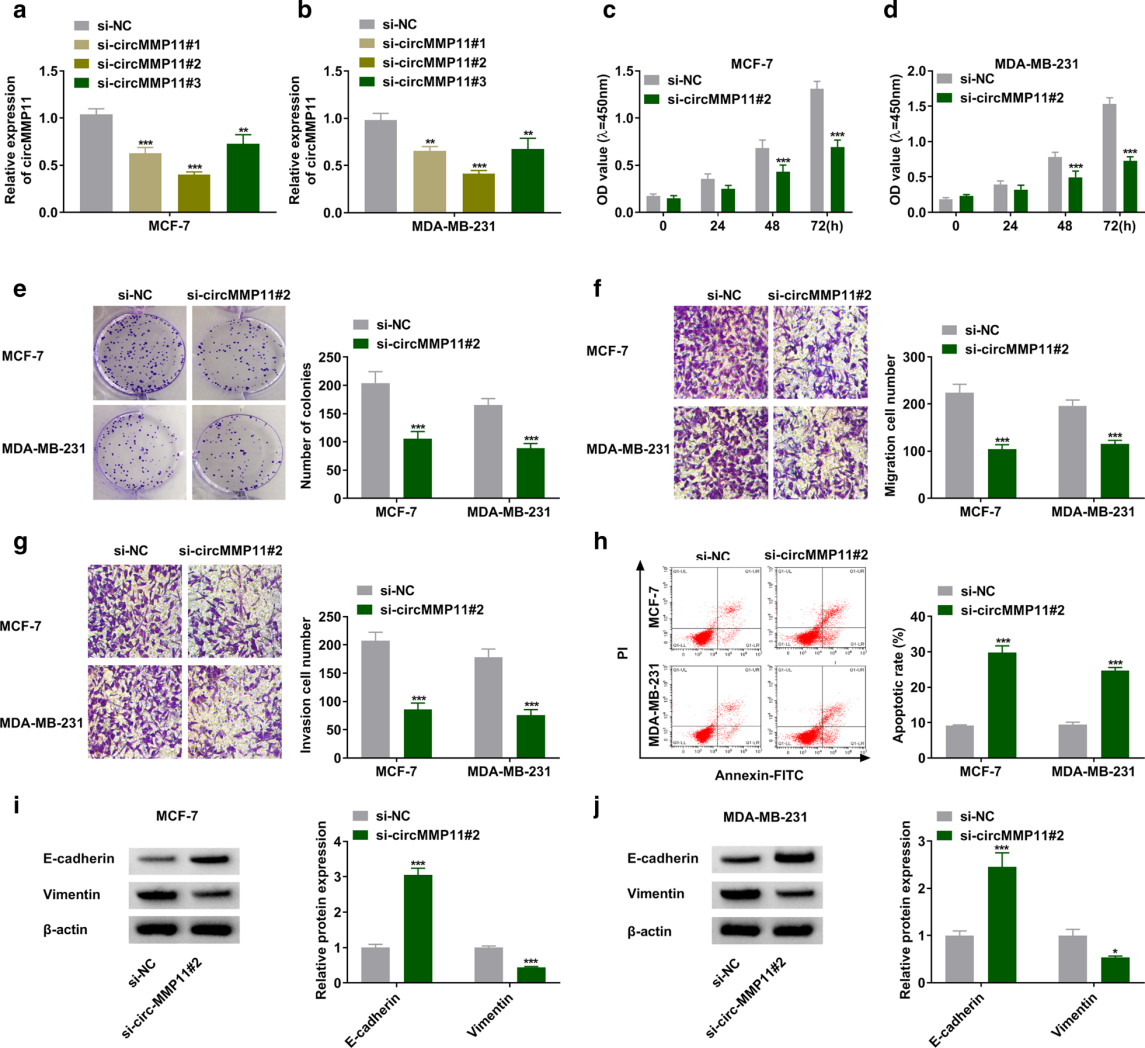
The effects of circMMP11 inhibition on proliferation, migration, invasion, and apoptosis of breast cancer cells. **a**, **b** The expression of circMMP11 was measured by RT-qPCR assay in MCF-7 and MDA-MB-231 cells transfected with si-NC, si-circMMP11#1, si-circMMP11#2, or si-circMMP11#3. **c**–**j** MCF-7 and MDA-MB-231 cells were transfected with si-NC or si-circMMP11#2. **c**–**e** The proliferation ability of MCF-7 and MDA-MB-231 cells were estimated by MTT and colony-forming assays. **f**, **g** Transwell assay was conducted to assess migration and invasion of MCF-7 and MDA-MB-231 cells. **h** The apoptotic cells were monitored by flow cytometry assay. **i**, **j** The protein expression levels of E-cadherin and Vimentin were quantified by western blot assay. **P < 0.01, ***P < 0.001

Subsequently, circMMP11 was inhibited by siRNAs targeting circMMP11 in MCF-7 and MDA-MB-231 cells. As shown in Fig. 2a, b, circMMP11 was downregulated in si-circMMP11 groups compared with control group, especially in si-circMMP11#2 group. MTT indicated suppression of circMMP11 decreased cell viability of MCF-7 and MDA-MB-231 cells (Fig. 2c, d). Likewise, colony-forming ability of BC cells was inhibited by silencing of circMMP11 (Fig. 2e). In addition, circMMP11 knockdown obviously suppressed migration and invasion of BC cells by transwell assay (Fig. 2f, g). We also observed that suppression of circMMP11 increased cell apoptosis (Fig. 2h). The analysis results of western blot assay indicated that inhibition of circMMP11 decreased Vimentin while increased E-cadherin expression in MCF-7 and MDA-MB-231 cells (Fig. 2i, j). Collectively, circMMP11 was aberrantly expressed as a tumor facilitator in BC.

### ZEB2 knockdown repressed proliferation, migration, and invasion while induced apoptosis of BC cells

**Fig. 3:**
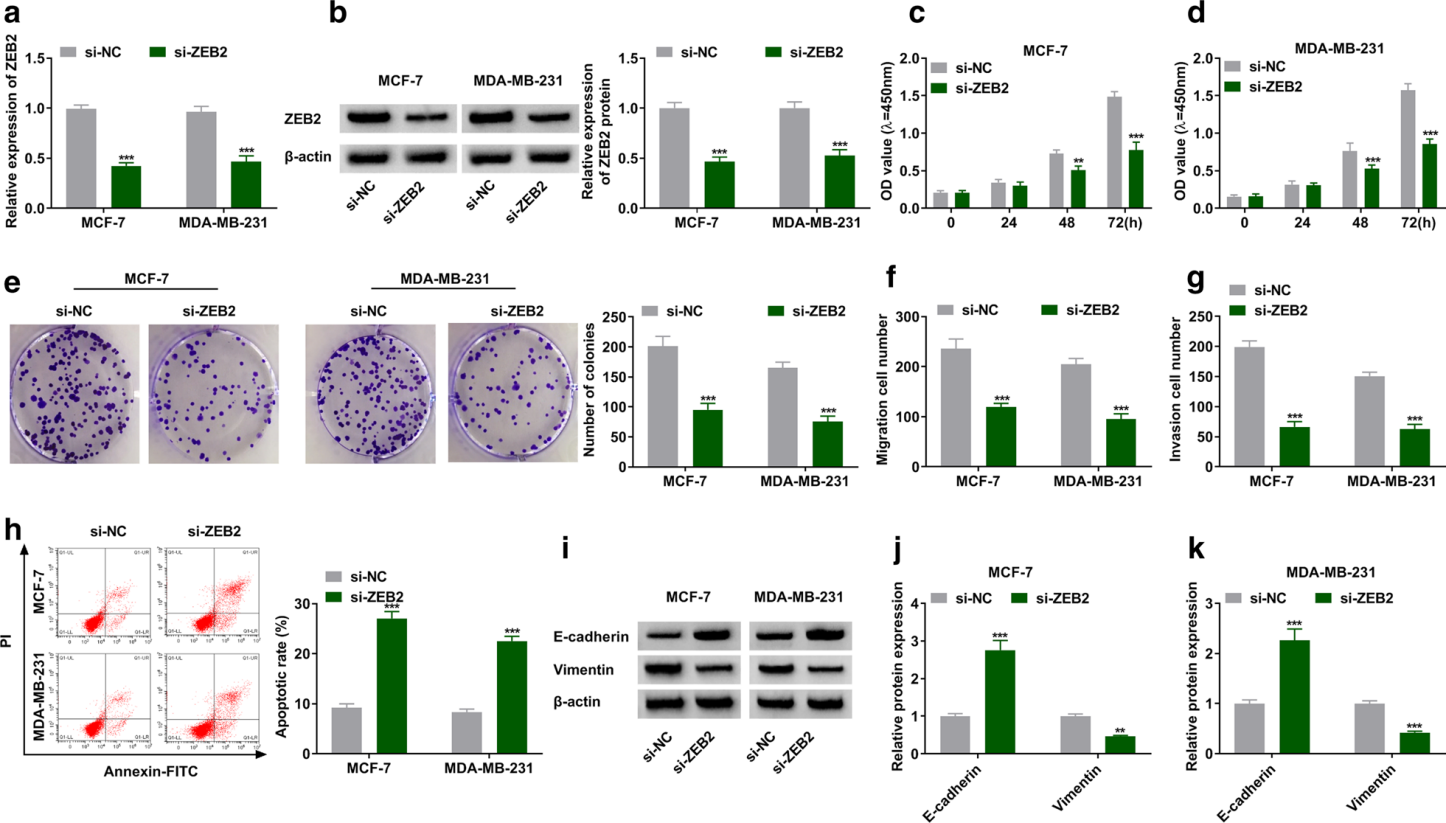
Suppression of ZEB2 inhibited proliferation, migration, and invasion while induced apoptosis of breast cancer cells. **a**–**k** MCF-7 and MDA-MB-231 cells were transfected with si-NC or si-ZEB2. **a**, **b** The expression of ZEB2 was detected by RT-qPCR assay in MCF-7 and MDA-MB-231 cells. **c**–**e** MTT and colony-forming assays were performed in MCF-7 and MDA-MB-231 cells. **f**, **g** The migration and invasion of MCF-7 and MDA-MB-231 cells were assessed by transwell assay. **h** The apoptosis was examined by flow cytometry assay. **i**–**k** The western blot assay was used to test protein expression of E-cadherin and Vimentin in MCF-7 and MDA-MB-231 cells. **P < 0.01, ***P < 0.001

Loss-of-function experiments were conducted in MCF-7 and MDA-MB-231 cells by siRNA targeting ZEB2. We found that ZEB2 was downregulated in MCF-7 and MDA-MB-231 cells after transfection with si-ZEB2 (Fig. 3a, b). Moreover, the inhibition of ZEB2 repressed the proliferation of MCF-7 and MDA-MB-231 cells (Fig. 3c–e). The reduced numbers of migration and invasion were found in si-ZEB2 transfection group compared with control group (Fig. 3f, g). The data of flow cytometry assay revealed that inhibition of ZEB2 enhanced cell apoptosis in BC cells (Fig. 3h). We also noticed that inhibition of ZEB2 increased the expression of E-cadherin while decreased the expression of Vimentin in MCF-7 and MDA-MB-231 cells (Fig. 3i, k). Therefore, these data indicated that silencing of ZEB2 might impede the development of BC.

### MiR-625-5p, interacted with ZEB2, was a target of circMMP11

**Fig. 4:**
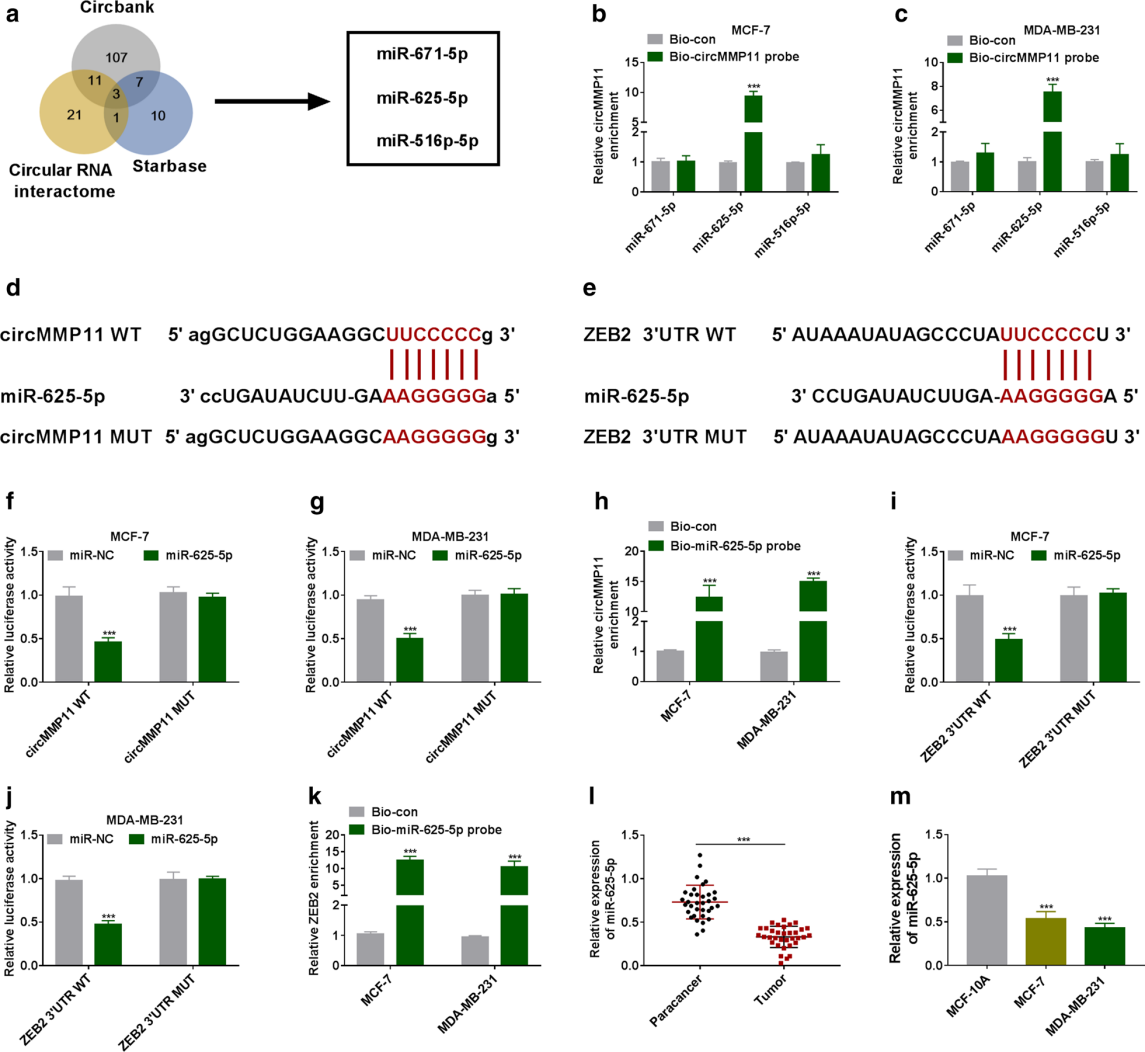
CircMMP11 increased ZEB2 expression through sponging miR-625-5p. **a** Schematic illustration showed the overlap of the target miRNAs of circMMP11 predicted by Circbank, Starbase, and Circular RNA interactome. **b**, **c** The relative levels of miRNA candidates in MCF-7 and MDA-MB-231 cells lysates were examined by RT-qPCR after pulling down by circMMP11 probe. **d** Predicted binding regions between circMMP11 and miR-625-5p were shown. **e** The binding sites for miR-625-5p in 3′UTR of ZEB2 were presented. **f**–**k** The interaction relationships among circMMP11, miR-625-5p, and ZEB2 were confirmed by dual-luciferase report and RNA pull-down assays. **l**, **m** The expression level of miR-625-5p was measured by RT-qPCR assay in breast cancer tissues and cells, along with in matched controls. ***P < 0.001

To investigate target miRNAs of circMMP11, bioinformatics software was used. As presented in Fig. 4a, the overlap of candidate miRNAs contained miR-671-5p, miR-625-5p, and miR-516p-5p. Interestingly, we found that biotin-labeled circMMP11 could effectively pull-down miR-625-5p in cell lysates from MCF-7 and MDA-MB-231 cells (Fig. 4b, c). The binding regions between miR-625-5p and circMMP11 or ZEB2 were presented in Fig. 4d, e. The luciferase activity was downregulated in the group of co-transfection of miR-625-5p mimic and circMMP11 WT in MCF-7 and MDA-MB-231 cells, while circMMP11 MUT group showed no notable change of luciferase activity (Fig. 4f, g). We also found that circMMP11 was enriched by biotin-labeled miR-625-5p probe, suggesting that circMMP11 could interact with miR-625-5p (Fig. 4h). Similarly, overexpression of miR-625-5p decreased the luciferase activity of ZEB2 3′UTR WT; besides, ZEB2 was upregulated in Bio-miR-625-5p probe group compared with control group (Fig. 4i, k). What’s more, miR-625-5p was decreased in BC tissues and cells compared with matched controls (Fig. 4l, m). All data indicated that circMMP11 regulated ZEB2 expression through sponging miR-625-5p.

### Overexpression of ZEB2 abolished circMMP11-mediated effects on BC cells

**Fig. 5:**
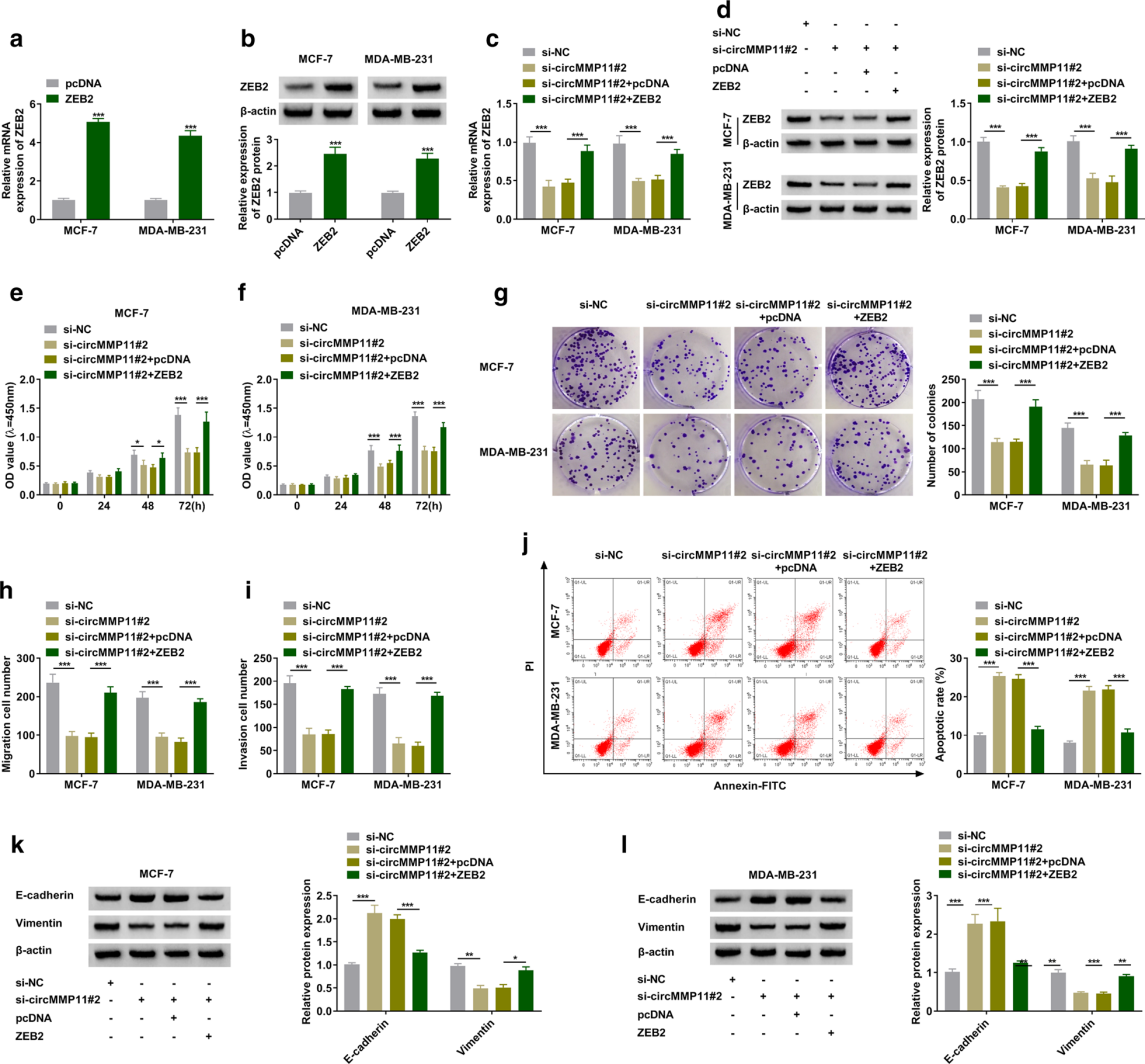
Knockdown of circMMP11-mediated effects on breast cancer cells could be abolished by overexpression of ZEB2. **a**, **b** The overexpression effectiveness of ZEB2 was checked by RT-qPCR and western blot assays. **c**–**l** MCF-7 and MDA-MB-231 cells were transfected with si-NC, si-circMMP11#2, si-circMMP11#2 + pcDNA, or si-circMMP11#2 + ZEB2. **c**, **d** The expression levels of ZEB2 were assessed by RT-qPCR and western blot assays. **e**–**g** The proliferation was examined by MTT and colony-forming assays in MCF-7 and MDA-MB-231 cells. **h**, **i** The migration and invasion were determined by transwell assay. **j** The flow cytometry assay was performed in MCF-7 and MDA-MB-231 cells. **i**–**k** The protein expression levels of E-cadherin and Vimentin were measured by western blot assay in MCF-7 and MDA-MB-231 cells. *P < 0.05, **P < 0.01, ***P < 0.001

The regulatory relationship between circMMP11 and ZEB2 was explored in MCF-7 and MDA-MB-231 cells. Western blot assay revealed that ZEB2 was obviously upregulated in MCF-7 and MDA-MB-231 cells after transfected with ZEB2 (Fig. 5a, b). The silencing of circMMP11 inhibited the expression of ZEB2, which was rescued by transfection with ZEB2 (Fig. 5c, d). The suppressive effect on cell proliferation in si-circMMP11#2-tranfecting cells was abolished by overexpression of ZEB2 (Fig. 5e, g). The migration and invasion of MCF-7 and MDA-MB-231 cells were suppressed by silencing of circMMP11, which was overturned by overexpression of ZEB2 (Fig. 5h, i). The upregulation of ZEB2 abolished circMMP11 inhibition-induced apoptosis in MCF-7 and MDA-MB-231 cells (Fig. 5j). The analysis results of western blot assay indicated that inhibition of circMMP11 decreased Vimentin while increased E-cadherin expression, which was overturned by overexpression of ZEB2 (Fig. 5k, l). Conclusively, circMMP11 knockdown repressed proliferation, migration, and invasion while induced apoptosis of BC cells through ZEB2.

### CircMMP11 regulated miR-625-5p/ZEB2 axis in BC

**Fig. 6:**
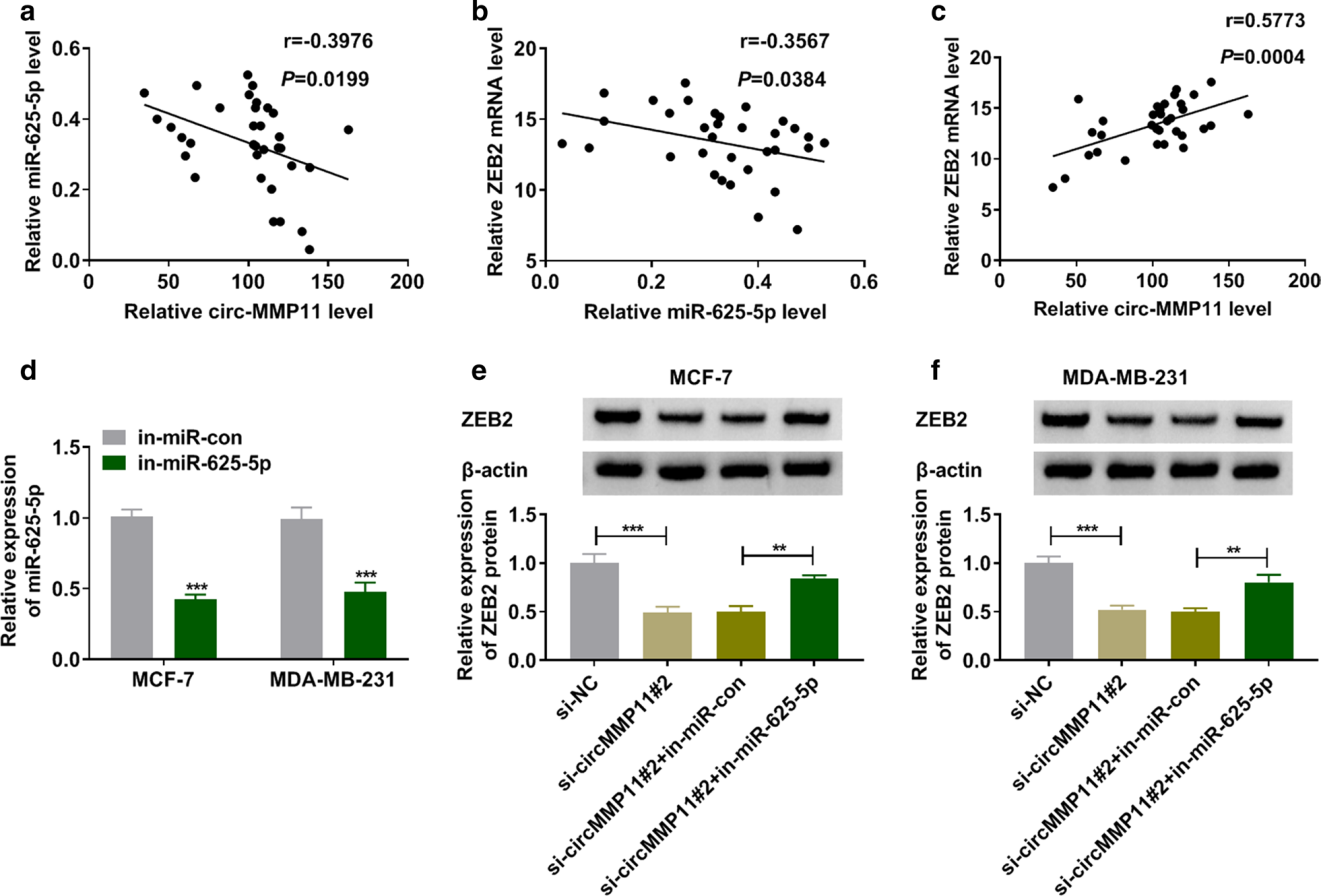
CircMMP11 regulated miR-625-5p/ZEB2 axis in breast cancer cells. **a**–**c** Pearson’s correlation analysis was used to reveal correlation relationship among circMMP11, miR-625-5p, and ZEB2 in breast cancer tissues. **d** The expression level of miR-625-5p was assessed by RT-qPCR in MCF-7 and MDA-MB-231 cells transfected with in-miR-con or in-miR-625-5p. **e**, **f** The expression levels of ZEB2 were measured by western blot assay in MCF-7 and MDA-MB-231 cells transfected with si-NC, si-circMMP11#2, si-circMMP11#2 + in-miR-co, or si-circMMP11#2 + in-miR-625-5p. **P < 0.01, ***P < 0.001

The correlation relationship among circMMP11, miR-625-5p, and ZEB2 was investigated in BC tissues. As presented in Fig. 6a, b, miR-625-5p was negatively correlated with circMMP11 and ZEB2 expression in BC tissues. Furthermore, a positive correlation between circMMP11 and ZEB2 was revealed in BC tissues (Fig. 6c). Transfection with in-miR-625-5p into MCF-7 and MDA-MB-231 cells inhibited the expression of miR-625-5p (Fig. 6d). The downregulation of ZEB2 in si-circMMP11#2-transfecing MCF-7 and MDA-MB-231 cells was abolished by inhibition of miR-625-5p (Fig. 6e, f). In summary, circMMP11 regulated ZEB2 expression in BC cells by targeting miR-625-5p.

### Inhibition of circMMP11 repressed tumorigenesis in vivo

**Fig. 7:**
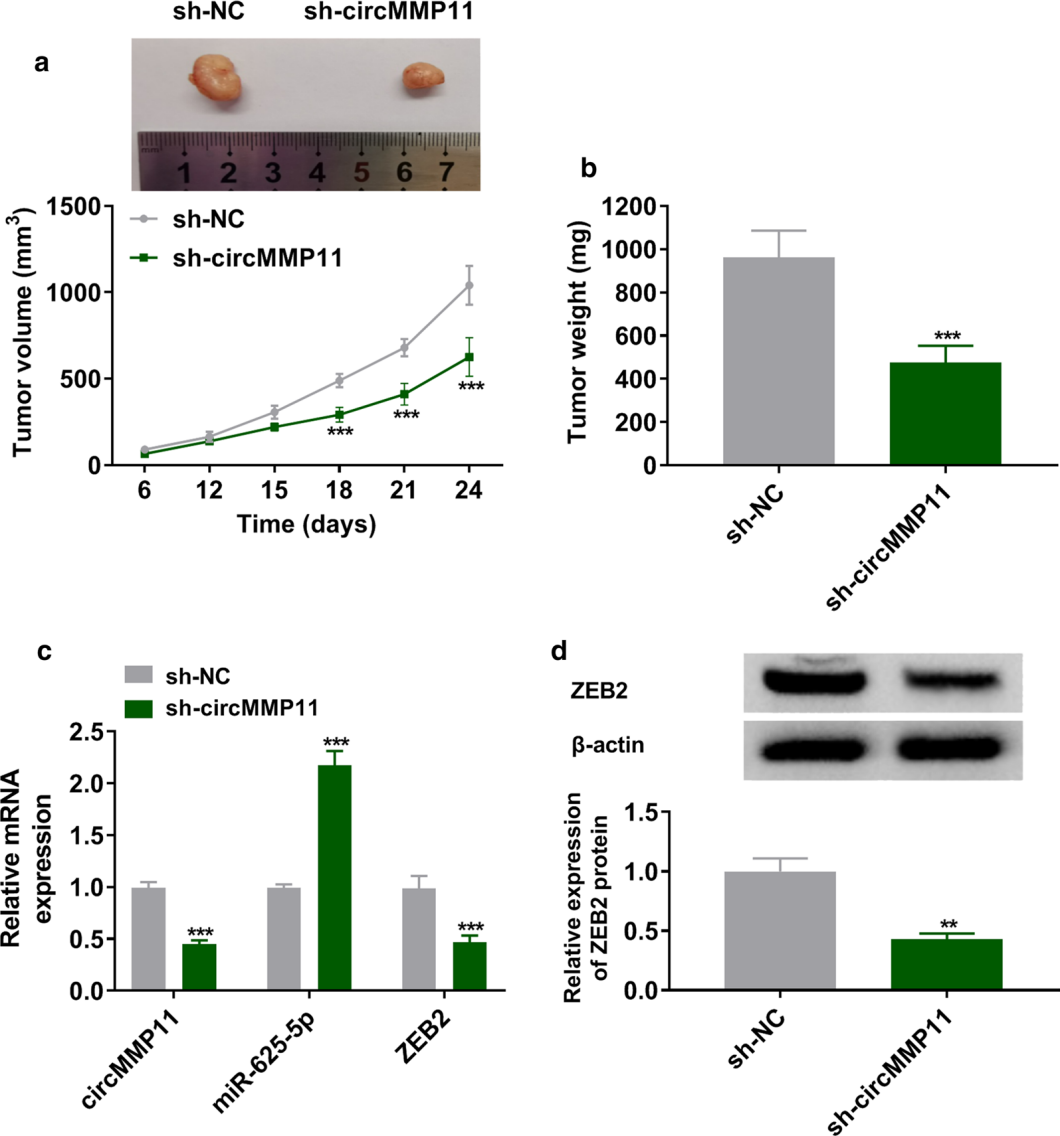
Silencing of circMMP11 repressed tumor growth in vivo. **a**, **b** The growth curves and weights of xenograft tumors were shown. **c** The expression levels of circMMP11, miR-625-5p, and ZEB2 were estimated with RT-qPCR assay in dissected tumor tissues. **d** Western blot assay was used to assess the expression level of ZEB2. **P < 0.01, ***P < 0.001

As shown in Fig. 7a, b, the knockdown of circMMP11 slowed the growth rate of tumor and decreased the weights of xenograft tumors when compared with control. The results of RT-qPCR assay indicated that circMMP11 and ZEB2 were decreased while miR-625-5p was increased in sh-circMMP11 group compared with sh-NC group (Fig. 7c). In addition, the suppression of circMMP11 also decreased the protein expression of ZEB2 in dissected tumor tissues (Fig. 7d). This finding confirmed that silencing of circMMP11 repressed tumor growth in vivo.

## Discussion

Currently, the regulatory networks of circRNA/microRNA/mRNA was widely focused and reported in tumorigenesis [16]. Therefore, the molecular mechanisms of circMMP11 was investigated in BC. Consistent with the previous conclusion [6, 7], our data indicated that circMMP11 was obviously higher in BC tissues than control group. In addition, suppression of circMMP11 suppressed cell growth and migration via directly targeting miR-625-5p/ZEB2 axis.

There are accumulating examples of miRNAs targeting mRNA, thereby participating the initiation and development of a large number of diseases, including BC [17]. Not surprisingly, a previous report implied that miR-625 was a possible and primary biomarker for BC [18]. MiR-625 played tumor-suppressive roles in malignant tumor; miR-625-5p could target LRRC8E and highmobilitygroupAT-hook1 (HMGA1) in cervical cancer and BC, respectively [19, 20]. Here, we demonstrated that miR-625-5p targeted ZEB2 in BC cells. Of course, other miRNAs could interact ZEB2 to regulate the occurrence and progression of BC; multiple miRNAs, including miR-30a [21], miR-653 [22], miR-448 [23], and miR-124 [24] were identified to bind to the 3′UTR of ZEB2 in BC cells.

Furthermore, the high metastasis and recurrence of BC impede clinical diagnosis, so inhibiting migration and invasion of BC cells is an important issue of current research [25]. EMT is one of the main mechanism, which induces metastasis and dispersion of malignant tumors and drug-resistance while prevents apoptosis [26]. During the EMT process, decrease of E-cadherin and increase of vimentin includes cellular mobility and invasion of tumor cells [27, 28]. Interestingly, ZEB2 exerts a crucial effect on the metastasis of malignancy cells due to its ability in stimulation of EMT process [29, 30]. Previous reports have indicated that ZEB was associated with EMT in retinoblastoma, which promoted the invasion and metastasis of retinoblastoma cells [31]. Our results also indicated that ZEB2 partially attenuated the suppressive influences of circMMP11 inhibition on cell growth and mobility of BC cells. In addition, ZEB2 was involved in EMT and downstream complex signaling pathways [32].

Nevertheless, certain limitations were faced during the study, including a limited sample size. In addition, cellular heterogeneity play the key roles in cancer management, application of protein expression assay at single cell level was necessary to build objective conclusion in the future works. Although our results suggested that the regulation of circMMP11/miR-625-5p/ZEB2 axis plays key roles in various biological functions of BC cells, the involved downstream signal paths and regulatory mechanisms have not been further investigated. Conclusively, our study provided evidence that circMMP11 was overexpressed in BC tissues and cells. CircMMP11 could directly bind to miR-625-5p, therefore promoting the expression of ZEB2 via ceRNA mechanism, which providing new insight into the pathogenesis and promising prognostic biomarker for BC.

## Conclusions

In summary, circMMP11 was obviously upregulated in BC tissues and cells. Mechanistic experiments suggested that circMMP11 exerted its tumor-promoting effects by modulating proliferation, migration, invasion, and apoptosis of BC cells through targeting miR-625-5p/ZEB2 axis, suggesting that circMMP11/miR-625-5p/ZEB2 axis could be used as diagnostic markers for BC.

## Data Availability

Author declares that all data and material are available by corresponding author.

## References

[1] Ghoncheh M, Pournamdar Z, Salehiniya H (2016). Incidence and mortality and epidemiology of breast cancer in the world. Asian Pac J Cancer Prev.

[2] Bray F, Ferlay J, Soerjomataram I, Siegel RL, Torre LA, Jemal A (2018). Global cancer statistics 2018: GLOBOCAN estimates of incidence and mortality worldwide for 36 cancers in 185 countries. CA Cancer J Clin.

[3] Pearce L (2016). Breast cancer. Nurs Stand.

[4] Patop IL, Kadener S (2018). circRNAs in cancer. Curr Opin Genet Dev.

[5] Kristensen LS, Andersen MS, Stagsted LVW, Ebbesen KK, Hansen TB, Kjems J (2019). The biogenesis, biology and characterization of circular RNAs. Nat Rev Genet.

[6] Li Z, Chen Z, Hu G, Zhang Y, Feng Y, Jiang Y (2020). Profiling and integrated analysis of differentially expressed circRNAs as novel biomarkers for breast cancer. J Cell Physiol.

[7] Li Z, Chen Z, Feng Y, Hu G, Jiang Y (2020). CircMMP11 acts as a ce-circRNA in breast cancer progression by regulating miR-1204. Am J Transl Res.

[8] Bartel DP (2004). MicroRNAs: genomics, biogenesis, mechanism, and function. Cell.

[9] Chen Z, Wu H, Zhang Z, Li G, Liu B (2019). LINC00511 accelerated the process of gastric cancer by targeting miR-625-5p/NFIX axis. Cancer Cell Int.

[10] Zhou WB, Zhong CN, Luo XP, Zhang YY, Zhang GY, Zhou DX (2016). miR-625 suppresses cell proliferation and migration by targeting HMGA1 in breast cancer. Biochem Biophys Res Commun.

[11] Wan Makhtar WR, Browne G, Karountzos A, Stevens C, Alghamdi Y, Bottrill AR (2017). Short stretches of rare codons regulate translation of the transcription factor ZEB2 in cancer cells. Oncogene.

[12] Zhang G, Li H, Sun R, Li P, Yang Z, Liu Y (2019). Long non-coding RNA ZEB2-AS1 promotes the proliferation, metastasis and epithelial mesenchymal transition in triple-negative breast cancer by epigenetically activating ZEB2. J Cell Mol Med.

[13] Duan Y, Zhang X, Yang L, Dong X, Zheng Z, Cheng Y (2019). Disruptor of telomeric silencing 1-like (DOT1L) is involved in breast cancer metastasis via transcriptional regulation of MALAT1 and ZEB2. J Genet Genomics.

[14] Chen X, Li J, Zhang S, Xu W, Shi D, Zhuo M (2019). MicroRNA30a regulates cell proliferation, migration, invasion and apoptosis in human nasopharyngeal carcinoma via targeted regulation of ZEB2. Mol Med Rep.

[15] You F, Luan H, Sun D, Cui T, Ding P, Tang H (2019). miRNA-106a promotes breast cancer cell proliferation, clonogenicity, migration, and invasion through inhibiting apoptosis and chemosensitivity. DNA Cell Biol.

[16] Rong D, Sun H, Li Z, Liu S, Dong C, Fu K (2017). An emerging function of circRNA-miRNAs-mRNA axis in human diseases. Oncotarget.

[17] Adhami M, Haghdoost AA, Sadeghi B, Malekpour Afshar R (2018). Candidate miRNAs in human breast cancer biomarkers: a systematic review. Breast Cancer.

[18] Si H, Sun X, Chen Y, Cao Y, Chen S, Wang H (2013). Circulating microRNA-92a and microRNA-21 as novel minimally invasive biomarkers for primary breast cancer. J Cancer Res Clin Oncol.

[19] Wang L, Zhong Y, Yang B, Zhu Y, Zhu X, Xia Z (2020). LINC00958 facilitates cervical cancer cell proliferation and metastasis by sponging miR-625-5p to upregulate LRRC8E expression. J Cell Biochem.

[20] Wu Z, Wang W, Wang Y, Wang X, Sun S, Yao Y (2020). Long noncoding RNA LINC00963 promotes breast cancer progression by functioning as a molecular sponge for microRNA-625 and thereby upregulating HMGA1. Cell Cycle.

[21] di Gennaro A, Damiano V, Brisotto G, Armellin M, Perin T, Zucchetto A (2019). Correction to: A p53/miR-30a/ZEB2 axis controls triple negative breast cancer aggressiveness. Cell Death Differ.

[22] Xie R, Tang J, Zhu X, Jiang H (2019). Silencing of hsa_circ_0004771 inhibits proliferation and induces apoptosis in breast cancer through activation of miR-653 by targeting ZEB2 signaling pathway. Biosci Rep.

[23] Ma P, Ni K, Ke J, Zhang W, Feng Y, Mao Q (2018). miR-448 inhibits the epithelial–mesenchymal transition in breast cancer cells by directly targeting the E-cadherin repressor ZEB1/2. Exp Biol Med (Maywood).

[24] Ji H, Sang M, Liu F, Ai N, Geng C (2019). miR-124 regulates EMT based on ZEB2 target to inhibit invasion and metastasis in triple-negative breast cancer. Pathol Res Pract.

[25] Warren M (2009). Metastatic breast cancer recurrence: a literature review of themes and issues arising from diagnosis. Int J Palliat Nurs.

[26] Lamouille S, Xu J, Derynck R (2014). Molecular mechanisms of epithelial–mesenchymal transition. Nat Rev Mol Cell Biol.

[27] Gupta GP, Massague J (2006). Cancer metastasis: building a framework. Cell.

[28] Katsuno Y, Lamouille S, Derynck R (2013). TGF-beta signaling and epithelial–mesenchymal transition in cancer progression. Curr Opin Oncol.

[29] Yoshimoto S, Tanaka F, Morita H, Hiraki A, Hashimoto S (2019). Hypoxia-induced HIF-1alpha and ZEB1 are critical for the malignant transformation of ameloblastoma via TGF-beta-dependent EMT. Cancer Med.

[30] Vandewalle C, Van Roy F, Berx G (2009). The role of the ZEB family of transcription factors in development and disease. Cell Mol Life Sci.

[31] Gao Y, Luo X, Zhang J (2020). Sp1-mediated up-regulation of lnc00152 promotes invasion and metastasis of retinoblastoma cells via the miR-30d/SOX9/ZEB2 pathway. Cell Oncol.

[32] Jeong MH, Kim HR, Park YJ, Chung KH (2019). Akt and Notch pathways mediate polyhexamethylene guanidine phosphate-induced epithelial–mesenchymal transition via ZEB2. Toxicol Appl Pharmacol.

